# Hyaluronidase: from clinical applications to molecular and cellular mechanisms

**DOI:** 10.1186/s40001-016-0201-5

**Published:** 2016-02-13

**Authors:** Bettina Alexandra Buhren, Holger Schrumpf, Norman-Philipp Hoff, Edwin Bölke, Said Hilton, Peter Arne Gerber

**Affiliations:** Department of Dermatology, Heinrich-Heine-University Düsseldorf, Düsseldorf, Germany; Department of Radiation Oncology, Medical Faculty, Heinrich-Heine-University Düsseldorf, Düsseldorf, Germany; Medical Skin Center Dr. Hilton & Partners, Düsseldorf, Germany

**Keywords:** Hyaluronidase, Hyaluronic acid, Hyaluronan, Filler, Dermatosurgery, Aesthetic dermatology, Adjuvant, Spreading effect, Diffusion promotor

## Abstract

Over the past 60 years, hyaluronidase has been successfully utilized in ophthalmic surgery and is now being implemented in dermatosurgery as well as in other surgical disciplines. The enzyme is considered a “spreading factor” as it decomplexes hyaluronic acid (also called hyaluronan, HA), an essential component of the extracellular matrix (ECM). When applied as an adjuvant, hyaluronidase enhances the diffusion capacity and bioavailability of injected drugs. Therefore, the enzyme has been used as a local adjuvant to increase the diffusion capacity of local anesthetics, increasing the analgesic efficacy, and the anesthetized area particularly in the first minutes following injection, resulting in diminished intra- and postoperative pain. In aesthetic medicine, the off-label use of hyaluronidase is considered the gold standard for the management of HA-filler-associated complications. Here, we review the clinical use, underlying biological mechanisms, and future directions for the application of hyaluronidase in surgical and aesthetic medicine.

## Background

### Regulation of hyaluronan (HA) metabolism

The extracellular matrix (ECM) of the skin consists of a complex and dynamic network of macromolecules. In addition to providing the structural framework, the ECM plays an important role in regulating various cellular mechanisms including proliferation, adhesion, migration, and gene regulation [1]. The main components of the ECM are the fibrous proteins collagen and elastin, as well as, the proteoglycans (PGs) to which characteristic glycosaminoglycan (GAG) chains are attached. The most common GAG in the dermis is hyaluronic acid (also called hyaluronan) [2]. Hyaluronan (HA) is a linear glycosaminoglycan disaccharide composed of alternating units of n-acteyl-d-glycosamine and d-glucuronic acid via alternating β-1.4 and β-1.3 glycosidic bonds [3]. Since it is extremely hydrophilic, HA has a high hydration capacity and, therefore, contributes to the viscoelastic properties of the skin.

Approximately 50 % of all HA in the body is found in skin tissue and with typical concentrations ranging from 0.5 to 1 mg/g in the dermis [3]. Unfortunately, HA content decreases with age, resulting in loss of skin moisture and laxity, both characteristic features of aging skin. Therefore, the cosmetic injection of HA-containing dermal fillers has evolved as one of the key strategies in skin rejuvenation.

Immunohistochemical analyses of biotinylated HA-binding proteins demonstrated that HA is not only synthesized by dermal fibroblasts but also by epidermal keratinocytes [4]. HA is synthesized by plasma membrane-bound enzymes, the HA synthase enzymes -1, -2, and -3 (HAS1-3), that extrude HA directly into the extracellular space. HAS1-3 exhibit distinct enzymatic properties and synthesize HA chains of various lengths [5].

The turnover of HA depends on location and has an approximate half-life in the skin of only one day or less. HA is degraded into smaller HA fragments (HAF) by hyaluronidases (HYAL) which hydrolyze the disaccharides at hexosaminidic β (1–4) linkages. Depending on the cleavage site, HAF size results in either oligosaccharides or other larger polymers. In addition, free radicals such as reactive oxygen species (ROS) can interact with HA leading to its degradation. In humans, six HYAL have been identified which contribute to this process: HYAL-1, -2, -3, -4, PH-20, and HYALP1 [6, 7]. PH-20 is a hyaluronidase, specific to the testes and is well known for its essential role in fertilization. Localized at the anterior sperm head surface, PH-20 facilitates the penetration of sperm through the cumulus cells that surround the oocyte embedded in an ECM which is rich in HA [8]. As testicular tissue is a physiological source of bioactive hyaluronidase, bovine hyaluronidase can be extracted from bovine testes.

The size of HA fragments can vary from high [HMW-HA; >4 × 10^5^ Da(lton)] and medium (MMW-HA; 5 × 10^4^–4 × 10^5^ Da) to low (LMW-HA; <5 × 10^4^ Da) molecular weights. Whereas HAS1 and HAS3 synthesize HA fragments of 2 × 10^5^ to 2 × 10^6^ Da in size, HAS2 generates HA fragments with approximate sizes of 2 × 10^6^ Da [9]. Interestingly, the molecular size of HA appears to be of critical importance for its various biological effects [10]. In its high molecular form, HA is generally a space-filling molecule that hydrates tissues. Moreover, HMW-HA has been shown to attenuate inflammatory responses as it has anti-inflammatory [11], anti-angiogenic [12], and also immunosuppressive properties [13], which suggests that it might play a role in promoting regenerative healing [14]. In addition, Tian et al. demonstrated that HMW-HA has intrinsic anti-aging and anti-cancer effects [15]. Conversely, LMW-HA has a proinflammatory activity. It has been found to be angiogenic [16], immunostimulatory [17], and inflammatory [18]. In addition, McKee et al. showed that LMW-HA increases the expression of macrophage inflammatory protein-1a and monocyte chemotactic protein-1, suggesting an important role in proinflammatory responses [19], as it may be an important step in the induction of the inflammatory cascade.

Hence, HA offers a wide functional spectrum and, considering the size-dependent effects, represents an important player in the regulation of skin homeostasis. However, the underlying mechanisms responsible for the size-dependent effects of HA fragments are not clearly understood.

In addition to humans, hyaluronidases have been found in a variety of venoms from snakes, lizards, and insects. In this capacity, they contribute to local damage and accelerate the spread of toxins at a bite site and affect the local integrity of the ECM due to degradation of HA [20]. Moreover, various species of gram positive bacteria (e.g., *Staphylococcus aureus*) are capable of producing bacterial HA lyases as a potential virulence factor to promote tissue penetration [21].

#### Clinical use

In Germany, bovine hyaluronidase (Hylase^®^ Dessau, Riemser Pharma GmbH, Greifswald, Germany) is approved as an adjuvant for infiltration anesthesia. By degrading HA in the ECM, hyaluronidase increases membrane permeability, thereby rendering tissues more permeable to injected fluids—the so-called spreading effect. As a consequence, hyaluronidase reduces viscosity of HA which improves tissue diffusion and the resorption rate of excess fluids. Co-administration of hyaluronidase is a potential option to enhance the effectiveness of local anesthesia; increasing the analgesic efficacy with respect to the anesthetized area per time (e.g., subcutaneous and intramuscular injections) [22]. Indeed, the therapeutic application of hyaluronidase has been established in a variety of surgical disciplines. In particular, hyaluronidase has been used in ophthalmic surgery for the past 60 years in combination with local anesthetics for peritubular, retrotubular or sub-Tenon’s anesthesia [23, 24] and is now being established in other surgical disciplines, including dermatosurgery.

The addition of hyaluronidase to local anesthetics in vitreoretinal surgery promotes the dispersion of the local anesthetics within the orbit by increasing the surrounding tissues permeability. Moreover, hyaluronidase minimizes the increasing orbital pressure associated with the volume of injected anesthetics and enhances the quality of globe akinesia which reduces the incidence of transient postoperative extraocular muscle paresis, as well as, the frequency of postoperative pain [25, 26]. In addition, it is proposed that the use of hyaluronidase improves the operation conditions of eyelid surgery, in particular for blepharoplasty [27].

Hyaluronidase treatment and application is generally well tolerated and adverse events are rare [28]. However, side effects from hyaluronidase use, such as temporary postinjection pruritus or allergic reactions, have been reported [22, 29]. When the influence of adjuvant hyaluronidase on wound healing was investigated by Wohlrab and colleagues using the suction blister method in a prospective, single-center, placebo-controlled, double-blind, intraindividual comparison study of 20 participants, no retardation of wound healing or other relevant risks were observed [22]. These clinical results are in line with own (mostly unpublished) in vitro wound-healing analyses, using primary human structural skin cells (primary human keratinocytes and dermal fibroblasts).

### Dermatosurgery

In dermatosurgery, the application of hyaluronidase is commonly used in regional block anesthesia and in a wide variety of other minor surgical procedures. Similar to findings from ophthalmologic procedures, the enzyme contributes to a higher bioavailability of the active substance within the target compartment compared to respective controls [29]. Reports on the use of hyaluronidase date back as far as 1951, when Thorpe noted the beneficial effects of hyaluronidase as an adjuvant to procaine in “Lancet” [30]. In 1994, Clark and Mellette [31] evaluated the effect of combining hyaluronidase with local anesthesia in 72 operations performed over a 1-year period. The authors concluded that benefits of the addition of hyaluronidase to local anesthesia included minimization of the loss of surface contour and enhanced ease in undermining and dissection through subcutaneous tissue planes [32]. In a prospective, randomized, placebo-controlled study, Wohlrab and colleagues reported that the co-application of hyaluronidase as a diffusion promotor of lidocaine in subcutaneous application resulted in an expanded effect in skin infiltration analgesia in 44 volunteers [33]. In 1998, Nevarre et al. showed that the area of anesthesia achieved by 1 % lidocaine infiltration can be significantly enhanced by the addition of hyaluronidase at a concentration of 15 IU/cc. The authors considered this effect to be attributable to the raised pH of the anesthetic area to a slightly more physiologic level, resulting in a pH closer to that of lidocaine pK and increasing its activity. Moreover, co-application of hyaluronidase significantly decreased tissue distortion without decreasing the efficacy of anesthetic action. Interestingly, when hyaluronidase was added to 1 % lidocaine, patients indicated significantly increased pain at the injection site—an aspect which had not been previously reported [33].

In routine clinical practice, hyaluronidase can be used as an additive to local anesthetics particularly for pain-sensitive areas such as the nasolabial fold, upper and lower eyelid, ear, lip, mucosa, anorectal, and perianal tissues, as well as, in nail surgery.

### Aesthetic dermatology

Progressive loss of dermal HA is one of the hallmarks of skin aging. The reasons for the decline in HA are a consequence of both, intrinsic and extrinsic factors, including reduced synthesis levels of HA in structural cells, for example, in fibroblasts (intrinsic aging) and a progressive degradation of HA due to exogenous stress factors such as repeated and extended exposure to UV radiation (extrinsic aging) [34, 35].

In addition to its use in local analgesia, the injection of HA-based fillers has become increasingly popular over the past several years and is now the preferred treatment for physicians performing soft tissue augmentations, deep skin hydration (native HA or “skin boosters”), or facial contouring. One major advantage of HA-based fillers, for example in contrast to calcium hydroxyl apatite-based (CaHA) or poly-l-lactic acid-based fillers (PLA), is the availability of a specific antidote, hyaluronidase, which is considered a rescue medication for the management of complications resulting from filler injections [36, 37].

Potential complications of aesthetic filler injections include overcorrections, the tyndall effect (bluish discoloration) or lower eyelid edema following tear-trough augmentation. Furthermore, granulomatous reactions, infections, visual impairment, or even blindness, as well as, local tissue necrosis caused by vascular occlusion as a result of intravascular HA injections or sidewall compression of vascular structures, can occur. In this context, the title of a review by Hirsch et al. underscores the importance of hyaluronidase for aesthetic or corrective dermatology: “Hyaluronidase in the office: a necessity for every dermatosurgeon that injects hyaluronic acid.” [38]

**Fig. 1:**
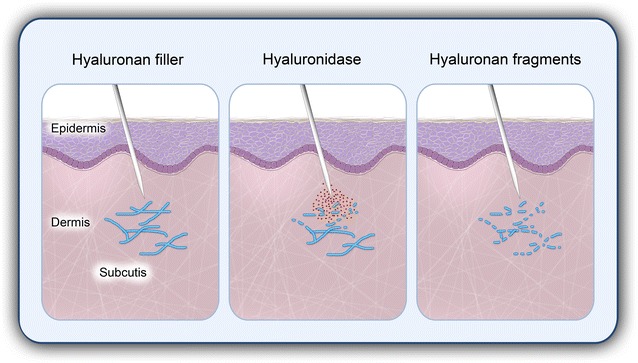
Hyaluronidase effectively degrades hyaluronan-based dermal fillers. The injection of hyaluronidase results in rapid degradation of the complex network of hyaluronan (HA)-fillers into HA fragments

Recently, we reported on the successful use of hyaluronidase to reverse a massive HA-injection-induced overcorrection of the glabellar rhytide performed by a nonmedical practitioner [39]. The infiltrative treatment with hyaluronidase resulted in complete remission within a few hours. Hence, hyaluronidase has the potency to effectively degrade HA-based fillers (Fig. 1). Whether this also accounts for all modern HA fillers that may be characterized by a high level of cross-linking in order to increase duration is currently under debate. Future studies may test the degradability of HA-based fillers by hyaluronidase in standardized in vitro and in vivo settings.

Besides overcorrection, the augmentation of the tear trough using HA fillers, even when performed by experienced physicians, is associated with an inherent risk for persistent lower eyelid edema which is correlated to the high water-binding capacity of HA. In a retroperspective analysis of patients presenting with edema following HA injection (tear-trough augmentation), we demonstrated that hyaluronidase (Hylase^®^ Dessau) injection effectively and rapidly resolved eyelid edema after a single injection [40]. Notably, early interventions (within a few weeks following development of edema) with hyaluronidase injection were more effective as compared to late interventions (after 6 months). For complete remission of chronic edema (persistent for more than 6 months), often multiple injections of hyaluronidase were required. In the clinical practice in respective cases, the injection of smaller volumes of hyaluronidase is recommended, as larger volumes may not only treat edema but may also degrade the effect of augmentation.

Facial regions prone to vascular side effects following dermal HA-filler injections correlate with the anatomy of superficial facial arteries. Respective “high risk areas” include the glabellar region (supratrochlear artery), the nasocilliar region (dorsal nasal artery), the temporal region (temporal arteries), the superior nasolabial fold and the tear trough (angular artery), or the nasolabial sulcus (facial artery). Early signs of vascular complications include immediate and severe pain as well as blanching due to the interruption of blood flow primarily beyond the site of injection. In the course, pain may increase and reddish to bluish discolorations of the skin may occur which may then progress to tissue necrosis. For the management of these complications, we recommend immediate infiltration of the entire area using large volumes of hyaluronidase. Hyaluronidase should not only be administered directly at the site of HA-filler injection, but also along the course of the obstructed artery. Importantly, direct intra-arterial injection of hyaluronidase is not necessary (or possible). Rather, perivascular hyaluronidase will permeate the vascular walls to decomplex intravascular HA. In 2011, Kim et al. evaluated whether early subcutaneous injections of hyaluronidase could decrease skin necrosis in HA-induced vascular complications [41]. Briefly, the authors used a rabbit ear model in which HA filler was injected into the auricular arteries. The authors evaluated the efficacy of early (<4 h after HA-filler injection) vs. late (24 h after HA-filler injection) infiltration of hyaluronidase. Interestingly, in the early intervention group hyaluronidase significantly reduced the size of necrotic areas compared to the late group. Therefore, it can be proposed that the injection of hyaluronidase for the management of vascular complications following HA-filler injections has a higher likelihood of success, when performed early (<4 h after HA-filler injection).

In conclusion, we propose the following recommendations for the use of hyaluronidase in aesthetic medicine:

- When working with dermal HA filler, hyaluronidase should always be immediately available [38].
- For aesthetic indications, hyaluronidase (Hylase^®^ Dessau) should be dissolved in 1.0 ml saline solution (0.9 % NaCl).
- Severe complications of vascular necrosis following accidental intravascular HA-filler injection should be immediately treated with infiltrations of large volumes of hyaluronidase in the entire area (ideally <4 h post HA-filler injection) [41].
- For the correction of HA overcorrections, the applied volume of hyaluronidase should not exceed the estimated volume of the overcorrection in order to avoid complete degradation of the effect of HA augmentation. Ideally, hyaluronidase should be injected gradually in small volumes and, when necessary, over multiple sessions in order to achieve the desired extent of correction and to prevent overtreatment [39].
- For the treatment of lower eyelid edema following HA augmentation of the tear trough, only a small volume of hyaluronidase should be applied at a time in order to only gradually dissolve excessive HA and to avoid complete reversal of the effect of HA augmentation [40].
- The efficacy of hyaluronidase treatment in the management of lower eyelid edema following HA augmentation of the tear trough is more effective when applied early (within weeks of the first appearance of edema) [40].

#### Open questions and perspectives

Today, the molecular and cellular mechanisms of HA catabolism within the ECM and in particular hyaluronidase-HA interactions have remained largely unknown. Frequently asked questions include: Does medical application of hyaluronidase influence postoperative wound healing and repair? Does clinical hyaluronidase-mediated HA degradation in the management of HA overcorrections also impair the skin´s physiological HA production? Does the application of hyaluronidase interfere with the natural aging process of the skin?

**Fig. 2:**
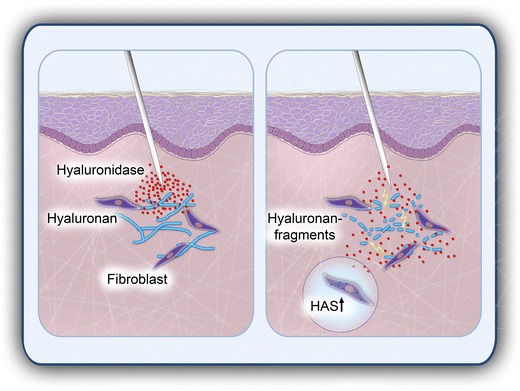
Degradation of dermal hyaluronan by infiltration of hyaluronidase. Dermal fibroblasts play a major role in neo-synthesis of hyaluronan (HA) as an essential component of the extracellular matrix (ECM). The injection of hyaluronidase degrades HA, and subsequently renders the ECM more permeable, resulting in a greater diffusion capacity and bioavailability of injected drugs. In addition, applied hyaluronidase induces the de novo synthesis of HA in dermal fibroblasts to compensate potential transient hyaluronidase-induced HA deficits

In this context, we can propose that our own, yet unpublished, in vitro experimental data show that bovine hyaluronidase (Hylase^®^ Dessau) does not negatively influence wound healing in a monolayer of primary human keratinocytes and primary human fibroblasts. In addition, we can propose that bovine hyaluronidase significantly and dose-dependently induces the synthesis of HA in structural skin cells. Degradation of body’s own HA by injection of hyaluronidase will therefore be immediately replaced by *de novo* synthesis of HA in fibroblasts (see Fig. 2). To what extent, however, the infiltration of excessive volumes of hyaluronidase may result in a temporary HA deficit due to a limited capacity of concurrent synthesis of HA remains a matter of debate. Clearly, in addition to excessive dermal HA filler, the exogenous administration of hyaluronidase will also decomplex naturally derived HA within the ECM.

Finally, besides its high potential in both surgery and aesthetic medicine, hyaluronidase has already been established in the management of extravasation of cytostatic drug infusion, as a therapeutic option in fibrotic diseases and for faster subcutaneous liquid absorption in pediatrics [29, 42]. In addition, subcutaneous application of monoclonal antibodies in combination with hyaluronidase also appears to be promising. Currently, the anti-HER2-antibody Trastuzumab (Herceptin^®^ SC) and anti-CD20-antibody Rituximab (MabThera^®^ SC) are used along with hyaluronidase as an excipient in subcutaneous (SC) formulations [43].

## Conclusion

In recent years, hyaluronidase has been well established in ophthalmic surgery as well as, in dermatosurgery. In aesthetic medicine, the use of hyaluronidase is considered as the gold standard for the management of complications of HA fillers and should be immediately available at every treatment. Further clinical and experimental studies may provide greater insights as to new and diverse therapeutic applications for hyaluronidase in clinical practice in the future.
